# *KRAS* Mutations Worsen Overall Survival Rates in Black but not White American Patients With Colon Cancer

**DOI:** 10.1016/j.gastha.2026.101068

**Published:** 2026-07-18

**Authors:** Minoru Koi, Susan Ahmedyar, Agnes Premkumar, Yuki Aisu, Temitope O. Keku, John M. Carethers

**Affiliations:** 1 Division of Gastroenterology and Hepatology, Department of Medicine, University of California San Diego, San Diego, California; 2 Department of Surgery, University of California San Diego, San Diego, California; 3 Center for Gastrointestinal Disease and Biology, University of North Carolina, Chapel Hill, North Carolina; 4 Moores Cancer Center, University of California San Diego, San Diego, California; 5 Herbert Wertheim School of Public Health and Human Longevity Science, University of California San Diego, San Diego, California

Both incidence and mortality rates from colorectal cancer (CRC) in Black American (BA) and White American (WA) populations were second and third highest, respectively, after American Indian and Alaskan Native groups in the United States.^1^ Despite recent decrease in disparity for incidence between BA and WA patients, there remains 1.25- to 1.4-fold higher CRC mortality disparity for BA patients.^2^ Multiple studies have shown the observed disparity emanates from differences in health-care access and treatment, comorbidities, and tumor characteristics.^2^, ^3^, ^4^ At the molecular level, higher prevalence of *KRAS* mutations and lower prevalence of microsatellite instability–high (MSI-H) in BA as compared to WA CRCs have been reported and may contribute to patient mortality differences;^5^, ^6^, ^7^, ^8^ it has rarely been determined how genetic changes such as *KRAS* mutations differentially impact patients’ outcomes across race. We previously demonstrated that loss of heterozygosity at chromosome 9p24.2 (9p24.2-LOH) and elevated microsatellite alterations at selected tetranucleotide repeats and/or microsatellite instability–low (E/L; induced by loss of the nuclear mismatch repair protein MSH3 in response to inflammation and/or hypoxia), were associated with recurrence and metastasis from primary CRC.^9^ Here, we determined whether and how genetic changes including *KRAS* mutations, *BRAF* mutations, MSI-H, 9p24.2-LOH, and E/L in colon cancers impact overall survival (OS) rate among 122 BA and 128 WA patients from the North Carolina Colon Cancer Study.^10^ Covariates including sex, age, tumor location, and tumor stage, comorbidities such as diabetes, high blood pressure, heart disease, and lifestyle factors including alcohol consumption and smoking habits, were also included for this analysis (see Supplementary Material and Methods).

We first identified independent variables associated with BA and WA colon cancer cases (Supplementary Table 1). Younger age (*P* = .01), higher consumption of alcohol (*P* = .003) and tobacco (*P* = .03), higher frequency of MSI-H (*P* = .01), and 9p24.2-LOH (*P* = .008) were associated with WA patients, while high blood pressure was associated with BA patients (*P* = .003) in multivariate analysis (Supplementary Table 1). For survival analysis, BA died faster than WA patients with colon cancer (hazard ratio [HR]: 1.67, 95% CI: 1.02–2.74, *P* = .04). *KRAS* mutation (HR: 2.0), older age (HR: 2.33), smoking (HR: 2.4), and higher tumor stage (HR: 4.96) but not tumor location (*P* = .06) were independently associated with lower 5-year OS (Figure 1A).

**Figure 1:**
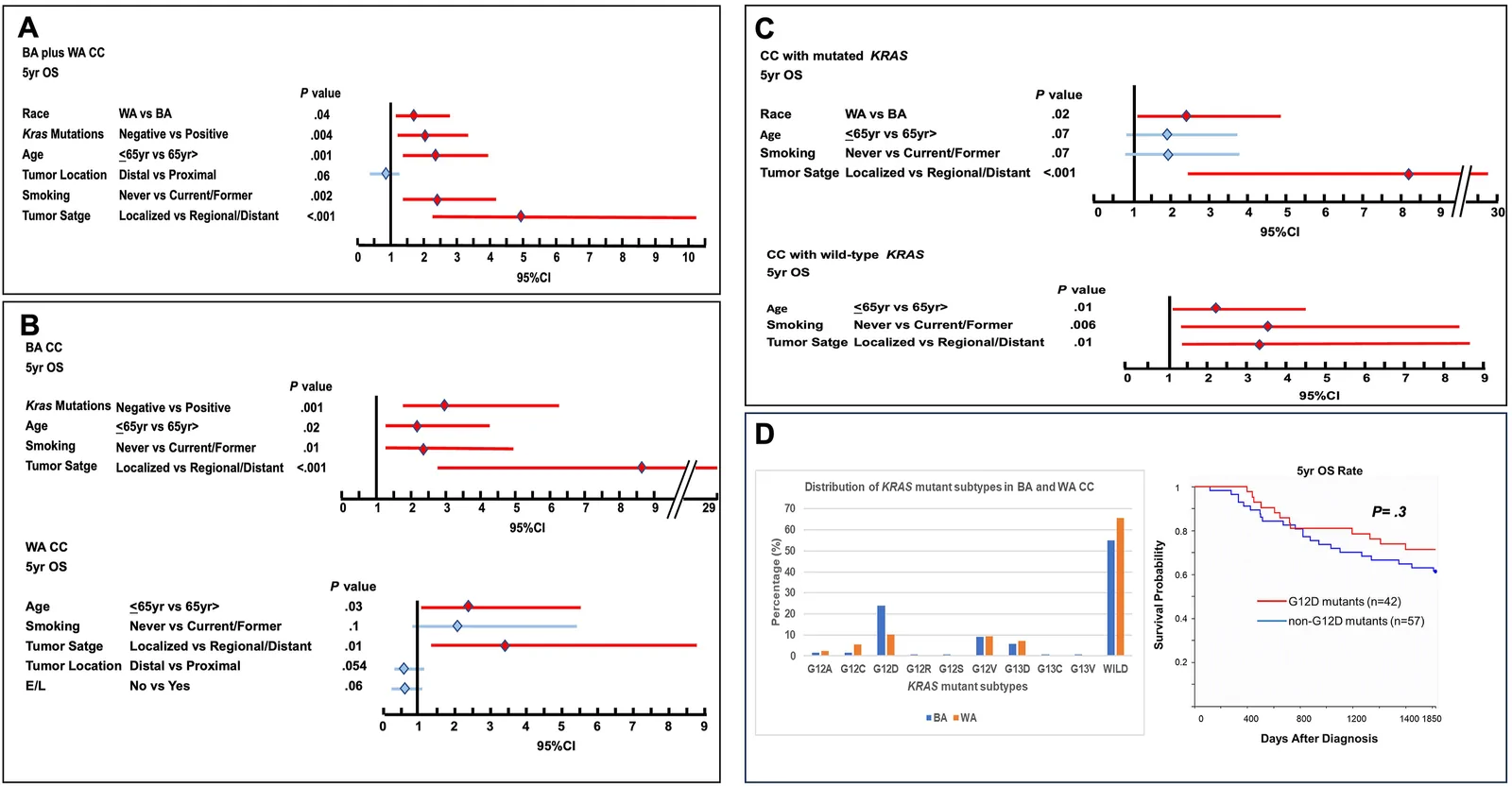
A, Cox proportional hazard test for 5-year OS of 250 colon cancer cases (122 BA and 128 WA). Each sample was analyzed for association between 5-year OS and each covariable including age (≤65 years: n = 125 vs >65 years: n = 125), sex (female: n = 116 vs male: n = 134), tumor location (distal: n = 96 vs proximal: n = 154) and stage (local: n = 85 vs regional/distant: n = 165), MSI-H status (negative: n = 225 vs positive: n = 25), EMAST status (negative: n = 138 vs positive: n = 112), *KRAS* G12G13 mutations (negative: n = 151 vs positive: n = 99), *BRAF*^V600E^ mutation (negative: n = 228 vs positive: n = 22), 9p24.2-LOH (negative: n = 195 vs positive: 55), alcohol use (no: n = 173 vs yes: n = 77), smoking (never: n = 106 vs current/former: n = 144), comorbid such as diabetes (no: n = 195 vs yes: n = 55), high blood pressure (no: n = 125 vs yes: n = 125), and heart disease (no: n = 186 vs yes: n = 64) using Cox regression analysis. The variables presented were the ones whose *P* values were less than 0.1 after multistep backward selection using SSPS software. The x-axis represents the range of the 95% confidence interval (CI). Each horizontal red (significant) or blue (nonsignificant) bar indicates 95% CI. The middle diamond shape represents the value for the hazard risk. The *P* value is shown following each variable. B, Upper panel: Cox proportional hazard test for 5-year OS of BA colon cancer cases (n = 122). Each sample was analyzed for 5-year OS and each covariable including age (≤65 years: n = 70 vs >65 years: n = 52), sex (female: n = 66 vs male: n = 56), tumor location (distal: n = 48 vs proximal: n = 74) and stage (local: n = 42 vs regional/distant: n = 80), MSI-H status (negative: n = 117 vs positive: n = 5), EMAST status (negative: n = 68 vs positive: n = 54), *KRAS* G12G13 mutations (negative: n = 67 vs positive: n = 55), *BRAF*^V600E^ mutation (negative: n = 113 vs positive: n = 9), 9p24.2-LOH (negative: n = 105 vs positive: 17), use of alcohol (no: n = 98 vs yes: n = 24), smoking (never: n = 65 vs current/former: n = 57), comorbidities such as diabetes (no: n = 92 vs yes: n = 30), high blood pressure (no: n = 50 vs yes: n = 72), and heart disease (no: n = 95 vs yes: n = 27) using Cox regression analysis. Lower panel: Cox proportional hazard test for 5-year OS of WA colon cancer cases (n = 128): Each sample was analyzed for 5-year OS and each covariable including age (≤65 years: n = 55 vs >65 years: n = 73), sex (female: n = 50 vs male: n = 78), tumor location (distal: n = 48 vs proximal: n = 80) and stage (local: n = 43 vs regional/distant: n = 85), MSI-H status (negative: n = 108 vs positive: n = 20), EMAST status (negative: n = 70 vs positive: n = 58), *KRAS* G12G13 mutations (negative: n = 84 vs positive: n = 44), *BRAF*^V600E^ mutation (negative: n = 115 vs positive: n = 13), 9p24.2-LOH (negative: n = 90 vs positive: 38), alcohol consumption (no: n = 75 vs yes: n = 53), smoking (never: n = 41 vs current/former: n = 87), comorbid such as diabetes (no: n = 103 vs yes: n = 25), high blood pressure (no: n = 75 vs yes: n = 53), and heart disease (no: n = 91 vs yes: n = 37) using Cox regression analysis. The variables presented were the ones whose *P* values were less than 0.1 after multistep backward selection using SSPS software. The x-axis represents the range of the 95% confidence interval (CI). Each horizontal red (significant) or blue (nonsignificant) bar indicates 95% CI. The middle diamond shape represents the value of the hazard risk. The *P* value is shown following each variable. C, Upper panel: Cox proportional hazard test for 5-year OS of colon cancers containing mutated *KRAS* (99 cases): Each sample was analyzed for 5-year OS and each covariable including race (white: n = 44 vs black: n = = 55), age (≤65 years: n = 51 vs >65 years: n = 48), sex (female: n = 46 vs male: n = 53), tumor location (distal: n = 34 vs proximal: n = 65) and stage (local: n = 33 vs regional/distant: n = 66), MSI-H status (negative: n = 95 vs positive: n = 4), EMAST status (negative: n = 58 vs positive: n = 41), *BRAF*^V600E^ mutation (negative: n = 97 vs positive: n = 2), 9p24.2-LOH (negative: n = 82 vs positive: 17), alcohol consumption (no: n = 31 vs yes: n = 68), smoking (never: n = 47 vs current/former: n = 52), comorbid such as diabetes (no: n = 81 vs yes: n = 18), high blood pressure (no: n = 48 vs yes: n = 51), and heart disease (no: n = 73 vs yes: n = 26) using Cox regression analysis. Lower panel: Cox proportional hazard test for 5-year OS of colon cancer containing wild-type *KRAS* (151 cases): Each sample was analyzed for 5-year OS and each covariable including race (white: n = 84 vs black: n = 67), age (≤65 years: n = 74 vs >65 years: n = 77), sex (female: n = 70 vs male: n = 81), tumor location (distal: n = 62 vs proximal: n = 89) and stage (local: n = 52 vs regional/distant: n = 99), MSI-H status (negative: n = 130 vs positive: n = 21), EMAST status (negative: n = 80 vs positive: n = 71), *BRAF*^V600E^ mutation (negative: n = 113 vs positive: n = 38), 9p24.2-LOH (negative: n = 113 vs positive: 38), alcohol consumption (no: n = 101 vs yes: n = 50), smoking (never: n = 59 vs current/former: n = 92), comorbidities such as diabetes (no: n = 114 vs yes: n = 37), high blood pressure (no: n = 77 vs yes: n = 74), and heart disease (no: n = 113 vs yes: n = 38) using Cox regression analysis. The variables presented were the ones whose *P* values were less than 0.1 after multistep backward selection using SSPS software. The x-axis represents the range of the 95% confidence interval (CI). Each horizontal red (significant) or blue (nonsignificant) bar indicates 95% CI. The middle diamond shape represents the value for the hazard risk. The *P* value is shown following each variable. D, Left panel: Frequency of *KRAS* mutation subtypes among BA and WA colon cancers: Ninety-nine colon cancers (BA: n = 55 and WA: n = 44) were found to contain mutations in *KRAS* codons 12 and 13. The percentage of cases with each mutant subtype among total BA (blue bar) and that of WA (red bar) is presented. The x-axis represents 9 different subtypes of *KRAS* mutations detected in BA and/or WA colon cancers. The Y-axis represents percent survival probability. Right panel: 5-year OS rate of colon cancer with *KRAS* G12D (n = 42) and colon cancers with other subtypes (n = 57) in Kaplan-Meier curves. Red line represents survival rate of patients with tumors containing *KRAS* G12D mutation, whereas blue line represents survival rate of patients with tumors containing other subtypes of *KRAS* G12G13 mutations. The x-axis represents days after diagnosis. The Y-axis represents percent survival probability. EMAST, elevated microsatellite alterations at selected tetranucleotide repeats.

We next determined risk factor(s) for 5-year OS among WA and BA patients with colon cancer. Among BA patients, *KRAS* mutations (HR: 2.98, 95% confidence interval [CI]: 1.54–5.79, *P* = .001) and other factors including age (HR: 2.15), smoking (HR: 2.37), and tumor stage (HR: 8.67) were associated with a lower 5-year OS (Figure 1B, upper panel). Among WA patients, age (HR: 2.37) and tumor stage (HR: 3.3) were associated with lower 5-year OS (Figure 1B, lower panel), while tumor location (*P* = .054), smoking (*P* = .1) or E/L (*P* = .06) were not risk factors. Importantly, in contrast to BA cases, *KRAS* mutations were not a risk factor for WA 5-year OS (HR: 1.3, 95% CI: 0.63–2.67, *P* = .47 by univariate Cox hazard analysis) (*data not shown*).

These results suggest that *KRAS* mutations worsen 5-year OS in BA but not in WA patients with colon cancer. To assess this hypothesis, we analyzed colon cancers with *KRAS* mutations (99 cases: 44 WA and 55 BA) and with wild-type (WT) *KRAS* (151 cases: 84 WA and 67 BA) for patient survival. Among patients whose tumors contain *KRAS* mutations, tumor stage (HR: 8.15) but not age (*P* = .07) nor smoking (*P* = .07) were risk factors for 5-year OS. Importantly, BA patients with *KRAS* mutations showed 2.33-fold faster rate of death within 5-years after diagnosis than WA patients with *KRAS* mutations (Figure 1C, upper panel). Among patients whose tumors contain WT *KRAS*, older age (HR: 2.53), smoking (HR: 3.45) and tumor stage (HR: 3.32) and were associated with a lower 5-year OS rate (Figure 1C bottom panel), while race was not a risk factor for 5-year OS in colon cancer with WT *KRAS* (HR: 0.96, 95% CI: 0.49–1.89, *P* = .91, univariate analysis, *not shown*). These results support the idea that effects of *KRAS* mutations on survival vary between BA and WA patients with colon cancer. Some of the disparity in survival due to *KRAS* mutations can be explained by higher frequency of *KRAS* mutations among BA patients.^6^, ^7^, ^8^ Although there was tendency for higher frequency of *KRAS* mutations in BA vs WA cases (45.1% vs 34.4%), this did not reach significance (odds ratio: 1.56, 95% CI: 0.91–2.61, *P* = .08, Supplementary Table 1). Because it has been reported that specific subtypes of *KRAS* mutations, especially *KRAS* G12D, is abundant in BA CRCs,^8^ we examined whether frequency of *KRAS* G12D was increased in our BA tumors and if it affected OS. As shown in the left panel of Figure 1D, *KRAS* G12D is the only subtype whose frequency was significantly increased in BA (29/122, 24%) compared to WA (13/128, 10.2%) cases (*P* = .03, Chi-square test). Furthermore, there was no evidence that patients with the *KRAS* G12D mutation experienced faster rate of death than patients with other subtypes (Figure 1D, right panel). These data indicate that neither increased frequency of *KRAS* mutations nor specific subtype of *KRAS* mutation is responsible for the observed survival disparity among BA cases. Finally, HR for race adjusted for age, tumor stage and location, and smoking with *KRAS* mutations was 1.67 (Figure 1A), while HR for race adjusted for the same variables without *KRAS* mutations was 1.71 (*not shown*). Based on these HRs, we estimate a disparity contribution by *KRAS* mutations of around 3.5% in 5-year OS between BA and WA patients.

Here, we demonstrate that among genetic alterations assessed in colon cancers, *KRAS* mutations are not only worse prognostic factors among BA but also contribute to the mortality disparity between BA and WA patients. The mechanism(s) for this disparity due to *KRAS* mutations is not clear; however, our results suggest that factor(s) other than frequency or subtype of *KRAS* mutations may be facilitators.

## Supplementary Materials

Supplementary Methods and Materials R1

Supplementary Table 1

Extended PDF
