# Supplementary material for: KRAS Mutations Worsen Overall Survival Rates in Black but not White American Patients With Colon Cancer
Source: Gastro Hep Adv. 2026 Jul 18;5(10):101068. doi: 10.1016/j.gastha.2026.101068 (PMC13499127; doi:10.1016/j.gastha.2026.101068)
Supplement: Supplementary file 1 [file mmc1.pdf]

KRAS Mutations Worsen Overall Survival Rates in Black but not White American Colon Cancer Patients

Minoru Koi[1], Susan Ahmedyar[1], Agnes Premkumar[2], Yuki Aisu[1], Temitope O. Keku[3], John M. Carethers[1,4,5]

[1] Division of Gastroenterology and Hepatology, Department of Medicine, University of California San Diego, San Diego, CA, USA
[2] Department of Surgery, University of California San Diego, San Diego, CA, USA
[3] Center for Gastrointestinal Disease and Biology, University of North Carolina, Chapel Hill, NC, USA
[4] Moores Cancer Center, University of California San Diego, San Diego, CA, USA
[5]Herbert Wertheim School of Public Health and Human Longevity Science, University of California San Diego

Supplementary Methods and Materials

Cohort description
The paired normal and tumor FFPE tissue samples from 128 White American and 122 Black American colon cancer cases were randomly chosen from the colon cancer cohort that has been previously characterized as patients who participated in the North Carolina Colon Cancer Study- Phase I (NCCCS I). Patients were between the ages of 40 to 80 years and resided in central North Carolina between 1996 and 2000. All tissues were obtained with informed consent and under IRB approval (1).

MSI/EMAST Assay
Paired normal and tumor genomic DNAs were extracted from formalin-fixed paraffin embedded (FFPE) tissues using the QIAamp DNA FFPE Tissue Kit (Qiagen). DNA was subjected to the PCR-based MSI/EMAST assay followed by fragment analysis. The assay consists of 14 microsatellite markers in four reactions capable of determining MSI-H, MSI-L/EMAST, and MSS status simultaneously. These markers include 2 mononucleotide (*BAT25* and *BAT26*), 5 dinucleotide (*D2S123*, *D5S346*, *D17S250*, *D18S64*, and *D18S69*) and 7 tetranucleotide microsatellite markers (*D9S242, D20S82, D20S85, D10S394, D8S321, MYCL1, RBM47*) (2).

KRAS(G12/G13) and BRAFV600E Mutation Detection
The mutational hot spots of *KRAS* codons 12 and 13 and *BRAF* codon 600 (valine to glutamic acid) were investigated by PCR-directed sequencing. PCR reactions were performed on tumor genomic DNA with the Q5 High Fidelity master mix (New England Biolaboratories) in a 96-well thermal cycler (Applied Biosystems). The primers used were: *KRAS* forward primer: 5′-GGTACTGGTGGAGTATTTGATAGTG-3′, *KRAS* reverse primer: 5′-ACCTCTATTGTTGGATCATATTCGT-3′, *BRAF* forward primer: 5′-TGCTTGCTCTGATAGGAAAATG-3′, and *BRAF*-reverse primer: 5′-AGTAACTCAGCAGCATCTCAGG-3′. PCR products were enzymatically purified with ExoSap-IT (Applied Biosystems) and subjected to Sanger sequencing (3,4).

9p24.2-LOH Detection
Loss of Heterozygosity (LOH) was determined at three SNP loci located at 9p24.2 region including rs16937852 (chr9;2177170, GRCh38/hg38, Allele: A/C), rs6475635 (chr9;2247072, Allele: C/T), and rs1002518 (chr9;2424322, Allele: C/T). Predesigned TaqMan based PCR assay for these SNPs were obtained from Thermo Fisher Scientific (Waltham, MA). To detect heterozygosity at the certain SNP locus, normal DNA from each sample was subjected to a real time PCR (Step One plus, Applied Biosystems) using the TaqMan based PCR assay. The amplified products were analyzed for heterozygosity using TaqMan Genotype Software (Thermo Fisher Scientific). To detect loss of heterozygosity at a certain locus, DNA from a tumor tissue whose normal counterpart exhibits heterozygosity at the locus was subjected to digital PCR (QuantStudio Absolute Q Digital PCR System, Thermo Fisher Scientific). We calculated LOH value as follows:

1-(smaller copy number from one allele / larger copy number from another allele) X100.

In our assay, LOH value never exceeds 10 at heterozygous SNP locus in normal tissue samples. We defined LOH-positive SNP when LOH values exceed 45. When a tumor exhibits LOH in at least one of three SNPs, we define the tumor as 9p24.2-LOH positive.

Statistics
All statistical analysis was completed using SPSS Software (Version 3, Chicago, IL). Logistic regression was used to investigate associations. Cox proportional hazard regression was used to determine risk factors for 5yr OS. In multivariate analysis, backward selection method (Backward LR: removes variables based on the probability of the likelihood-ratio) was used. Removal criteria were set to $p=0.1$. A p-value of less than 0.05 was deemed to be significant. Kaplan-Meier curve coupled with log-rank test was used for survival comparison. Chi-square test was used to determine relationships.

Data Availability
The data discussed in this study is available upon request from the corresponding author.

References

1. Satia JA, Keku T, Galanko JA, Martin C, Doctolero RT, Tajima A, Sandler RS, Carethers JM. Cancer Diet, lifestyle, and genomic instability in the North Carolina Colon Cancer Study. Epidemiol Biomarkers Prev. 2005;14(2):429-36.

2. Munakata K, Koi M, Kitajima T, et al. Inflammation-Associated Microsatellite Alterations Caused by MSH3 Dysfunction Are Prevalent in Ulcerative Colitis and Increase with Neoplastic Advancement. *Clin Transl Gastroenterol*. 2019;10(12):e00105. doi:10.14309/ctg.0000000000000105

3. Okita Y, Koi M, Takeda K, et al. Fusobacterium nucleatum infection correlates with two types of microsatellite alterations in colorectal cancer and triggers DNA damage. *Gut Pathog*. 2020;12(1):46. doi:10.1186/s13099-020-00384-3

4. Takeda K, Koi M, Okita Y, Sajibu S, Keku TO, Carethers JM. *Fusobacterium nucleatum* Load Correlates with *KRAS* Mutation and Sessile Serrated Pathogenesis in Colorectal Adenocarcinoma. *Cancer Research Communications*. 2023 Sep 26;3(9):1940-1951. doi: 10.1158/2767-9764.CRC-23-0179.
