# Supplementary material for: KRAS Mutations Worsen Overall Survival Rates in Black but not White American Patients With Colon Cancer
Source: Gastro Hep Adv. 2026 Jul 18;5(10):101068. doi: 10.1016/j.gastha.2026.101068 (PMC13499127; doi:10.1016/j.gastha.2026.101068)
Supplement: Supplementary file 2 [file mmc2.pdf]

Supplemetary Table 1

Characterization of BA and WA Colon Cancer Cohort

Variables		No. of Cases (%) White	No. of Cases (%) Black	Univariate White vs Black O.R.	Univariate White vs Black 95%CI	Univariate White vs Black *p*	Multivariate White vs Black O.R.	Multivariate White vs Black 95%CI	Multivariate White vs Black *p*
Race		128	122	N.A.	N.A.	N.A.	N.A.	N.A.	N.A.
Age	≤65	55 (43)	70 (57.3)						
	>65	73 (57)	52 (42.7)	0.56	0.33-0.92	0.02	0.48	0.27-0.85	0.01
Sex	Female	50 (39)	66 (54.1)						
	Male	78 (61)	56 (45.9)	0.54	0.32-0.89	0.01	-	-	-
Tumor Locatior	Distal	48 (37.5)	48 (39.3)						
	Proximal	80 (62.5)	74 (60.7)	0.92	0.55-1.54	0.76	-	-	-
Tumor Stage	Local	43 (33.6)	42 (34.4)						
	Regional/Distant	85 (66.4)	80 (65.6)	0.96	0.57-1.62	0.89	-	-	-
MSI-H	No	108 (84.4)	117 (96)						
	Yes	20 (15.6)	5 (4)	0.23	0.08-0.63	0.005	0.25	0.08-0.75	0.01
EMAST /MSI-L	No	70 (54.7)	68 (55.7)						
	Yes	58 (45.3)	54 (44.3)	0.95	0.58-1.57	0.86	-	-	-
KRAS G12/G1:	No	84 (65.6)	67 (54.9)						
Mutations	Yes	44 (34.4)	55 (45.1)	1.56	0.91-2.61	0.08	-	-	-
BRAF V600E	No	115 (89.8)	113 (92.6)						
	Yes	13 (10.2)	9 (7.4)	0.7	0.29-1.71	0.44	-	-	-
9p24.2-LOH	No	90 (70.3)	105 (86.1)						
	Yes	38 (29.7)	17 (13.9)	0.38	0.2-0.72	0.003	0.39	0.19-0.78	0.008
Alcohol drink	No	75 (58.6)	98 (80.3)						
	Yes	53 (41.4)	24 (19.7)	0.34	0.19-0.61	<0.001	0.38	0.2-0.73	0.003
Smoking	Never	41 (32)	65 (53.2)						
	Current/Former	87 (68)	57 (46.8)	0.41	0.24-0.69	<0.001	0.53	0.3-0.94	0.03
Diabetes	No	103 (84.4)	92 (75.4)						
	Yes	25 (15.6)	30 (24.6)	1.34	0.73-2.45	0.33	-	-	-
High Blood Pre	No	75 (58.6)	50 (41)						
	Yes	53 (41.4)	72 (59)	2.03	1.23-3.37	0.006	2.46	1.35-4.49	0.003
Heart Problem	No	91 (71.1)	95 (77.9)						
	Yes	37 (28.9)	27 (22.1)	0.69	0.39-1.24	0.22	0.52	0.26-1.03	0.06

Association between race and other covariates was determined via univariate and multivariate logistic regression analysis.

In multivariate analysis, covariates were subjected to multiple backward selection untill all of the remaining covariates exhibit less than 0.1 p-value using SPSS software.

- : not included in final calculations

N.A.: not applicable
