# Supplementary material for: KRAS Mutations Worsen Overall Survival Rates in Black but not White American Patients With Colon Cancer
Source: Gastro Hep Adv. 2026 Jul 18;5(10):101068. doi: 10.1016/j.gastha.2026.101068 (PMC13499127; doi:10.1016/j.gastha.2026.101068)
Supplement: Supplementary file 3 [file mmc3.pdf]

Gastro Hep Advances 2026;5:101068

RESEARCH LETTERS

KRAS Mutations Worsen Overall Survival Rates in Black but not White American Patients With Colon Cancer

Both incidence and mortality rates from colorectal cancer (CRC) in Black American (BA) and White American (WA) populations were second and third highest, respectively, after American Indian and Alaskan Native groups in the United States.[1] Despite recent decrease in disparity for incidence between BA and WA patients, there remains 1.25- to 1.4-fold higher CRC mortality disparity for BA patients.[2] Multiple studies have shown the observed disparity emanates from differences in health-care access and treatment, comorbidities, and tumor characteristics.[2–4] At the molecular level, higher prevalence of *KRAS* mutations and lower prevalence of microsatellite instability–high (MSI-H) in BA as compared to WA CRCs have been reported and may contribute to patient mortality differences;[5–8] it has rarely been determined how genetic changes such as *KRAS* mutations differentially impact patients' outcomes across race. We previously demonstrated that loss of heterozygosity at chromosome 9p24.2 (9p24.2-LOH) and elevated microsatellite alterations at selected tetranucleotide repeats and/or microsatellite instability–low (E/L; induced by loss of the nuclear mismatch repair protein MSH3 in response to inflammation and/or hypoxia), were associated with recurrence and metastasis from primary CRC.[9] Here, we determined whether and how genetic changes including *KRAS* mutations, *BRAF* mutations, MSI-H, 9p24.2-LOH, and E/L in colon cancers impact overall survival (OS) rate among 122 BA and 128 WA patients from the North Carolina Colon Cancer Study.[10] Covariates including sex, age, tumor location, and tumor stage, comorbidities such as diabetes, high blood pressure, heart disease, and lifestyle factors including alcohol consumption and smoking habits, were also included for this analysis (see Supplementary Material and Methods).

We first identified independent variables associated with BA and WA colon cancer cases (Supplementary Table 1). Younger age (P = .01), higher consumption of alcohol (P = .003) and tobacco (P = .03), higher frequency of MSI-H (P = .01), and 9p24.2-LOH (P = .008) were associated with WA patients, while high blood pressure was associated with BA patients (P = .003) in multivariate analysis (Supplementary Table 1). For survival analysis, BA died faster than WA patients with colon cancer (hazard ratio [HR]: 1.67, 95% CI: 1.02–2.74, P = .04). *KRAS* mutation (HR: 2.0), older age (HR: 2.33), smoking (HR: 2.4), and higher tumor stage (HR: 4.96) but not tumor location (P = .06) were independently associated with lower 5-year OS (Figure 1A).

We next determined risk factor(s) for 5-year OS among WA and BA patients with colon cancer. Among BA patients, *KRAS* mutations (HR: 2.98, 95% confidence interval [CI]: 1.54–5.79, P = .001) and other factors including age (HR: 2.15), smoking (HR: 2.37), and tumor stage (HR: 8.67) were associated with a lower 5-year OS (Figure 1B, upper panel). Among WA patients, age (HR: 2.37) and tumor stage (HR: 3.3) were associated with lower 5-year OS (Figure 1B, lower panel), while tumor location (P = .054), smoking (P = .1) or E/L (P = .06) were not risk factors. Importantly, in contrast to BA cases, *KRAS* mutations were not a risk factor for WA 5-year OS (HR: 1.3, 95% CI: 0.63–2.67, P = .47 by univariate Cox hazard analysis) (*data not shown*).

These results suggest that *KRAS* mutations worsen 5-year OS in BA but not in WA patients with colon cancer. To assess this hypothesis, we analyzed colon cancers with *KRAS* mutations (99 cases: 44 WA and 55 BA) and with wild-type (WT) *KRAS* (151 cases: 84 WA and 67 BA) for patient survival. Among patients whose tumors contain *KRAS* mutations, tumor stage (HR: 8.15) but not age (P = .07) nor smoking (P = .07) were risk factors for 5-year OS. Importantly, BA patients with *KRAS* mutations showed 2.33-fold faster rate of death within 5-years after diagnosis than WA patients with *KRAS* mutations (Figure 1C, upper panel). Among patients whose tumors contain WT *KRAS*, older age (HR: 2.53), smoking (HR: 3.45) and tumor stage (HR: 3.32) and were associated with a lower 5-year OS rate (Figure 1C bottom panel), while race was not a risk factor for 5-year OS in colon cancer with WT *KRAS* (HR: 0.96, 95% CI: 0.49–1.89, P = .91, univariate analysis, *not shown*). These results support the idea that effects of *KRAS* mutations on survival vary between BA and WA patients with colon cancer. Some of the disparity in survival due to *KRAS* mutations can be explained by higher frequency of *KRAS* mutations among BA patients.[6–8] Although there was tendency for higher frequency of *KRAS* mutations in BA vs WA cases (45.1% vs 34.4%), this did not reach significance (odds ratio: 1.56, 95% CI: 0.91–2.61, P = .08, Supplementary Table 1). Because it has been reported that specific subtypes of *KRAS* mutations, especially *KRAS* G12D, is abundant in BA CRCs,[8] we examined whether frequency of *KRAS* G12D was increased in our BA tumors and if it affected OS. As shown in the left panel of Figure 1D, *KRAS* G12D is the only subtype whose frequency was significantly increased in BA (29/122, 24%) compared to WA (13/128, 10.2%) cases (P = .03, Chi-square test). Furthermore, there was no evidence that patients with the *KRAS* G12D mutation experienced faster rate of death than patients with other subtypes (Figure 1D, right panel). These data indicate that neither increased frequency of *KRAS* mutations nor specific subtype of *KRAS* mutation is responsible for the observed survival disparity among BA cases. Finally, HR for race adjusted for age, tumor stage and location, and smoking with *KRAS*

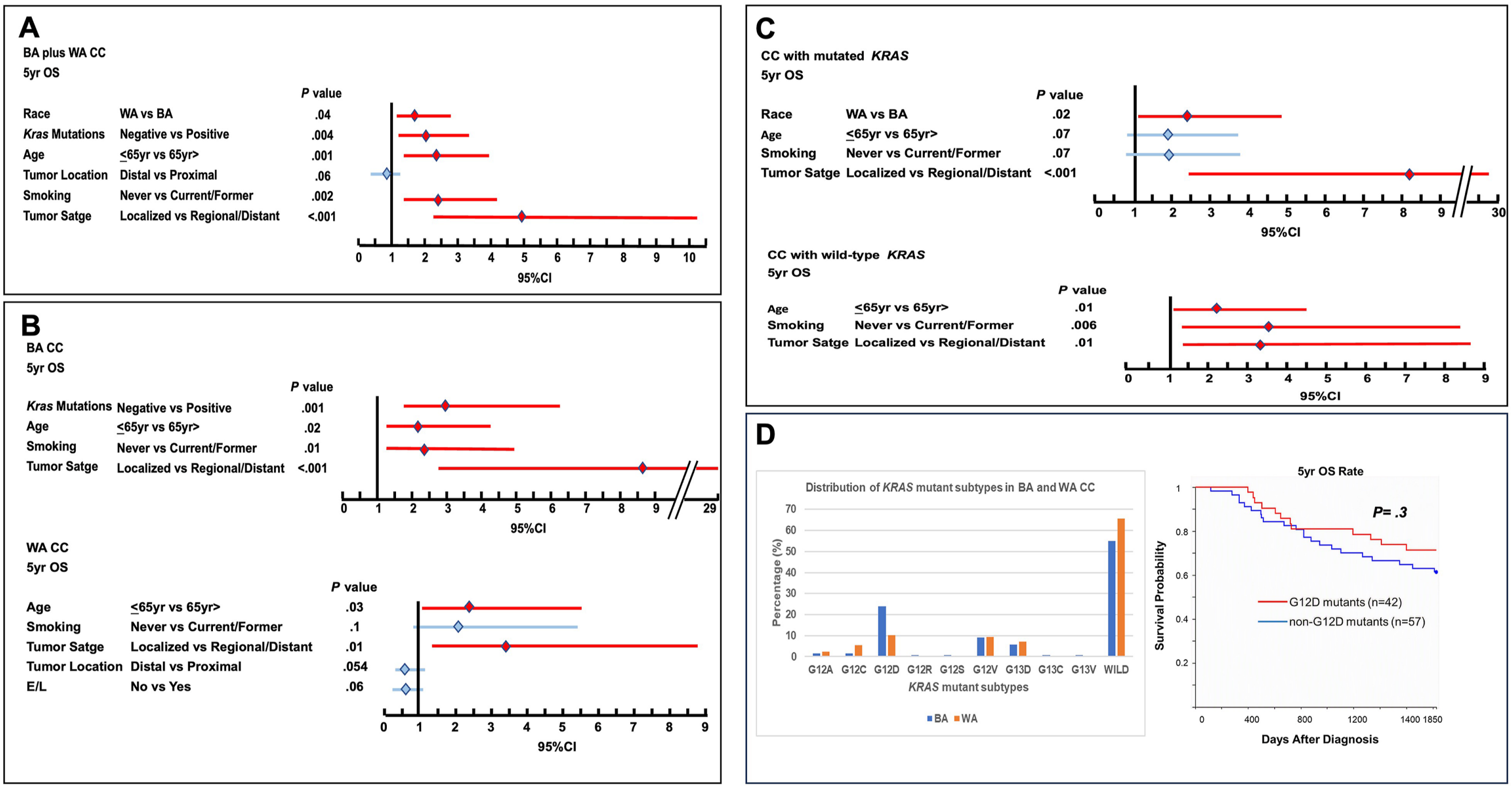

Figure 1. A, Cox proportional hazard test for 5-year OS of 250 colon cancer cases (122 BA and 128 WA). Each sample was analyzed for association between 5-year OS and each covariable including age (≤65 years: n = 125 vs >65 years: n = 125), sex (female: n = 116 vs male: n = 134), tumor location (distal: n = 96 vs proximal: n = 154) and stage (local: n = 85 vs regional/distant: n = 165), MSI-H status (negative: n = 225 vs positive: n = 25), EMAST status (negative: n = 138 vs positive: n = 112), *KRAS* G12G13 mutations (negative: n = 151 vs positive: n = 99), *BRAF*^{V600E} mutation (negative: n = 228 vs positive: n = 22), 9p24.2-LOH (negative: n = 195 vs positive: 55), alcohol use (no: n = 173 vs yes: n = 77), smoking (never: n = 106 vs current/former: n = 144), comorbid such as diabetes (no: n = 195 vs yes: n = 55), high blood pressure (no: n = 125 vs yes: n = 125), and heart disease (no: n = 186 vs yes: n = 64) using Cox regression analysis. The variables presented were the ones whose *P* values were less than 0.1 after multistep backward selection using SSPS software. The x-axis represents the range of the 95% confidence interval (CI). Each horizontal red (significant) or blue (nonsignificant) bar indicates 95% CI. The middle diamond shape represents the value for the hazard risk. The *P* value is shown following each variable. B, Upper panel: Cox proportional hazard test for 5-year OS of BA colon cancer cases (n = 122). Each sample was analyzed for 5-year OS and each covariable including age (≤65 years: n = 70 vs >65 years: n = 52), sex (female: n = 66 vs male: n = 56), tumor location (distal: n = 48 vs proximal: n = 74) and stage (local: n = 42 vs regional/distant: n = 80), MSI-H status (negative: n = 117 vs positive: n = 5), EMAST status (negative: n = 68 vs positive: n = 54), *KRAS* G12G13 mutations (negative: n = 67 vs positive: n = 55), *BRAF*^{V600E} mutation (negative: n = 113 vs positive: n = 9), 9p24.2-LOH (negative: n = 105 vs positive: 17), use of alcohol (no: n = 98 vs yes: n = 24), smoking (never: n = 65 vs current/former: n = 57), comorbidities such as diabetes (no: n = 92 vs yes: n = 30), high blood pressure (no: n = 50 vs yes: n = 72), and heart disease (no: n = 95 vs yes: n = 27) using Cox regression analysis. Lower panel: Cox proportional hazard test for 5-year OS of WA colon cancer cases (n = 128): Each sample was analyzed for 5-year OS and each covariable including age (≤65 years: n = 55 vs >65 years: n = 73), sex (female: n = 50 vs male: n = 78), tumor location (distal: n = 48 vs proximal: n = 80) and stage (local: n = 43 vs regional/distant: n = 85), MSI-H status (negative: n = 108 vs positive: n = 20), EMAST status (negative: n = 70 vs positive: n = 58), *KRAS* G12G13 mutations (negative: n = 84 vs positive: n = 44), *BRAF*^{V600E} mutation (negative: n = 115 vs positive: n = 13), 9p24.2-LOH (negative: n = 90 vs positive: 38), alcohol consumption (no: n = 75 vs yes: n = 53), smoking (never: n = 41 vs current/former: n = 87), comorbid such as diabetes (no: n = 103 vs yes: n = 25), high blood pressure (no: n = 75 vs yes: n = 53), and heart disease (no: n = 91 vs yes: n = 37) using Cox regression analysis. The variables presented were the ones whose *P* values were less than 0.1 after multistep backward selection using SSPS software. The x-axis represents the range of the 95% confidence interval (CI). Each horizontal red (significant) or blue (nonsignificant) bar indicates 95% CI. The middle diamond shape represents the value of the hazard risk. The *P* value is shown following each variable. C, Upper panel: Cox proportional hazard test for 5-year OS of colon cancers containing mutated *KRAS* (99 cases): Each sample was analyzed for 5-year OS and each covariable including race (white: n = 44 vs black: n = = 55), age (≤65 years: n = 51 vs >65 years: n = 48), sex (female: n = 46 vs male: n = 53), tumor location (distal: n = 34 vs proximal: n = 65) and stage (local: n = 33 vs regional/distant: n = 66), MSI-H status (negative: n = 95 vs positive: n = 4), EMAST status (negative: n = 58 vs positive: n = 41), *BRAF*^{V600E} mutation (negative: n = 97 vs positive: n = 2), 9p24.2-LOH (negative: n = 82 vs positive: 17), alcohol consumption (no: n = 31 vs yes: n = 68), smoking (never: n = 47 vs current/former: n = 52), comorbid such as diabetes (no: n = 81 vs yes: n = 18), high blood pressure (no: n = 48 vs yes: n = 51), and heart disease (no: n = 73 vs yes: n = 26) using Cox regression analysis. Lower panel: Cox proportional hazard test for 5-year OS of colon cancer containing wild-type *KRAS* (151 cases): Each sample was analyzed for 5-year OS and each covariable including race (white: n = 84 vs black: n = 67), age (≤65 years: n = 74 vs >65 years: n = 77), sex

mutations was 1.67 (Figure 1A), while HR for race adjusted for the same variables without *KRAS* mutations was 1.71 (*not shown*). Based on these HRs, we estimate a disparity contribution by *KRAS* mutations of around 3.5% in 5-year OS between BA and WA patients.

Here, we demonstrate that among genetic alterations assessed in colon cancers, *KRAS* mutations are not only worse prognostic factors among BA but also contribute to the mortality disparity between BA and WA patients. The mechanism(s) for this disparity due to *KRAS* mutations is not clear; however, our results suggest that factor(s) other than frequency or subtype of *KRAS* mutations may be facilitators.

MINORU KOI[1]
SUSAN AHMEDYAR[1]
AGNES PREMKUMAR[2]
YUKI AISU[1]
TEMITOPE O. KEKU[3]
JOHN M. CARETHERS[1,4,5]
[1]Division of Gastroenterology and Hepatology, Department of Medicine, University of California San Diego, San Diego, California
[2]Department of Surgery, University of California San Diego, San Diego, California
[3]Center for Gastrointestinal Disease and Biology, University of North Carolina, Chapel Hill, North Carolina
[4]Moores Cancer Center, University of California San Diego, San Diego, California
[5]Herbert Wertheim School of Public Health and Human Longevity Science, University of California San Diego, San Diego, California

Correspondence:
Address correspondence to: John M. Carethers, Office of the Vice Chancellor for Health Sciences, Biomedical Sciences Building Rm 1318A, 9500 Gilman Drive, La Jolla, California 92093-0602. e-mail: jcarethers@ucsd.edu.

Supplementary Materials

Material associated with this article can be found, in the online version, at https://doi.org/10.1016/j.gastha.2026.101068.

References

1. Siegel RL, et al. CA Cancer J Clin 2026;76(2):e70067.
2. Saka AH, et al. CA Cancer J Clin 2025;75(2):111–140.
3. Carethers JM. Adv Cancer Res 2021;151:197–229.
4. Koi M, et al. PLoS One 2020;15(10):e0239676.
5. Carethers JM, et al. PLoS One 2014;9(6):e100461.
6. Rodriguez GM, et al. JAMA Netw Open 2026;9(3):e261585.
7. Lawler T, et al. Cancer Causes Control 2024;35(2):223–239.
8. Myer PA, et al. Cancer Discov 2022;12(5):1282–1293.
9. Koi M, et al. Gastroenterology 2016;150(4):944–955.
10. Satia JA, et al. Cancer Epidemiol Biomarkers Prev 2005;14(2):429–436.

Abbreviations used in this paper: **9p24.2-LOH, loss of heterozygosity at chromosome 9p24.2; BA, Black American; CRC, colorectal cancer; E/L, elevated microsatellite alterations at selected tetranucleotide repeats and/or microsatellite instability–low; HR, hazard ratio; MSI-H, microsatellite instability–high; OS, overall survival; WA, White American; WT, wild-type**

Most current article

© 2026 The Authors. Published by Elsevier Inc. on behalf of American Gastroenterological Association Institute. This is an open access article under the CC BY license (http://creativecommons.org/licenses/by/4.0/).
2772-5723
https://doi.org/10.1016/j.gastha.2026.101068

Received April 20, 2026. Accepted July 14, 2026.

Authors' Contributions:
Minoru Koi: Study concept and design; acquisition of data, methodology, and investigation; technical and material support; analysis and interpretation of data; writing of manuscript; critical revision of manuscript for important intellectual content; statistical analysis; and study supervision and project administration and resources. Susan Ahmedyar: Acquisition of data, methodology, and investigation and technical and material support. Agnes Premkumar: Analysis and interpretation of data, critical revision of manuscript for important intellectual content, and statistical analysis. Yuki Aisu: Critical revision of manuscript for important intellectual content. Temitope O. Keku: Technical and material support. John M. Carethers: Technical and material support, analysis and interpretation of data, obtained funding, critical revision of manuscript for important intellectual content, and study supervision and project administration and resources.

Conflicts of Interest:
The authors disclose no conflicts.

Funding:
This work was supported in part by the United States Public Health Service (R01 CA258519 and T32CA275782) and funds from the University of California San Diego. The funders had no role in study design, data collection and analysis, decision to publish, or preparation of the manuscript.

Ethical Statement:
Samples were previously collected under institutional review board approval at the University of North Carolina School of Medicine and equivalent committees at the collaborating hospitals (see Supplementary Material and Methods and reference #10).

Data Transparency Statement:
The authors confirm that the data supporting the findings of this study are available in the article and/or its supplementary materials. The authors agree to make data and materials supporting the results or analyses presented in the paper available upon reasonable request.

Reporting Guidelines:
CoBRA.

(female: n = 70 vs male: n = 81), tumor location (distal: n = 62 vs proximal: n = 89) and stage (local: n = 52 vs regional/distant: n = 99), MSI-H status (negative: n = 130 vs positive: n = 21), EMAST status (negative: n = 80 vs positive: n = 71), $BRAF^{V600E}$ mutation (negative: n = 113 vs positive: n = 38), 9p24.2-LOH (negative: n = 113 vs positive: 38), alcohol consumption (no: n = 101 vs yes: n = 50), smoking (never: n = 59 vs current/former: n = 92), comorbidities such as diabetes (no: n = 114 vs yes: n = 37), high blood pressure (no: n = 77 vs yes: n = 74), and heart disease (no: n = 113 vs yes: n = 38) using Cox regression analysis. The variables presented were the ones whose P values were less than 0.1 after multistep backward selection using SSPS software. The x-axis represents the range of the 95% confidence interval (CI). Each horizontal red (significant) or blue (nonsignificant) bar indicates 95% CI. The middle diamond shape represents the value for the hazard risk. The P value is shown following each variable. D, Left panel: Frequency of KRAS mutation subtypes among BA and WA colon cancers: Ninety-nine colon cancers (BA: n = 55 and WA: n = 44) were found to contain mutations in KRAS codons 12 and 13. The percentage of cases with each mutant subtype among total BA (blue bar) and that of WA (red bar) is presented. The x-axis represents 9 different subtypes of KRAS mutations detected in BA and/or WA colon cancers. The Y-axis represents percent survival probability. Right panel: 5-year OS rate of colon cancer with KRAS G12D (n = 42) and colon cancers with other subtypes (n = 57) in Kaplan-Meier curves. Red line represents survival rate of patients with tumors containing KRAS G12D mutation, whereas blue line represents survival rate of patients with tumors containing other subtypes of KRAS G12G13 mutations. The x-axis represents days after diagnosis. The Y-axis represents percent survival probability. EMAST, elevated microsatellite alterations at selected tetranucleotide repeats.

Gastro Hep Advances, Volume *5*

Supplemental information

KRAS Mutations Worsen Overall Survival Rates in Black but not White American Patients With Colon Cancer

Minoru Koi, Susan Ahmedyar, Agnes Premkumar, Yuki Aisu, Temitope O. Keku, and John M. Carethers

KRAS Mutations Worsen Overall Survival Rates in Black but not White American Colon Cancer Patients

Minoru Koi[1], Susan Ahmedyar[1], Agnes Premkumar[2], Yuki Aisu[1], Temitope O. Keku[3], John M. Carethers[1,4,5]

[1] Division of Gastroenterology and Hepatology, Department of Medicine, University of California San Diego, San Diego, CA, USA
[2] Department of Surgery, University of California San Diego, San Diego, CA, USA
[3] Center for Gastrointestinal Disease and Biology, University of North Carolina, Chapel Hill, NC, USA
[4] Moores Cancer Center, University of California San Diego, San Diego, CA, USA
[5]Herbert Wertheim School of Public Health and Human Longevity Science, University of California San Diego

Supplementary Methods and Materials

Cohort description
The paired normal and tumor FFPE tissue samples from 128 White American and 122 Black American colon cancer cases were randomly chosen from the colon cancer cohort that has been previously characterized as patients who participated in the North Carolina Colon Cancer Study- Phase I (NCCCS I). Patients were between the ages of 40 to 80 years and resided in central North Carolina between 1996 and 2000. All tissues were obtained with informed consent and under IRB approval (1).

MSI/EMAST Assay
Paired normal and tumor genomic DNAs were extracted from formalin-fixed paraffin embedded (FFPE) tissues using the QIAamp DNA FFPE Tissue Kit (Qiagen). DNA was subjected to the PCR-based MSI/EMAST assay followed by fragment analysis. The assay consists of 14 microsatellite markers in four reactions capable of determining MSI-H, MSI-L/EMAST, and MSS status simultaneously. These markers include 2 mononucleotide (*BAT25* and *BAT26*), 5 dinucleotide (*D2S123*, *D5S346*, *D17S250*, *D18S64*, and *D18S69*) and 7 tetranucleotide microsatellite markers (*D9S242, D20S82, D20S85, D10S394, D8S321, MYCL1, RBM47*) (2).

KRAS(G12/G13) and BRAFV600E Mutation Detection
The mutational hot spots of *KRAS* codons 12 and 13 and *BRAF* codon 600 (valine to glutamic acid) were investigated by PCR-directed sequencing. PCR reactions were performed on tumor genomic DNA with the Q5 High Fidelity master mix (New England Biolaboratories) in a 96-well thermal cycler (Applied Biosystems). The primers used were: *KRAS* forward primer: 5′-GGTACTGGTGGAGTATTTGATAGTG-3′, *KRAS* reverse primer: 5′-ACCTCTATTGTTGGATCATATTCGT-3′, *BRAF* forward primer: 5′-TGCTTGCTCTGATAGGAAAATG-3′, and *BRAF*-reverse primer: 5′-AGTAACTCAGCAGCATCTCAGG-3′. PCR products were enzymatically purified with ExoSap-IT (Applied Biosystems) and subjected to Sanger sequencing (3,4).

9p24.2-LOH Detection
Loss of Heterozygosity (LOH) was determined at three SNP loci located at 9p24.2 region including rs16937852 (chr9;2177170, GRCh38/hg38, Allele: A/C), rs6475635 (chr9;2247072, Allele: C/T), and rs1002518 (chr9;2424322, Allele: C/T). Predesigned TaqMan based PCR assay for these SNPs were obtained from Thermo Fisher Scientific (Waltham, MA). To detect heterozygosity at the certain SNP locus, normal DNA from each sample was subjected to a real time PCR (Step One plus, Applied Biosystems) using the TaqMan based PCR assay. The amplified products were analyzed for heterozygosity using TaqMan Genotype Software (Thermo Fisher Scientific). To detect loss of heterozygosity at a certain locus, DNA from a tumor tissue whose normal counterpart exhibits heterozygosity at the locus was subjected to digital PCR (QuantStudio Absolute Q Digital PCR System, Thermo Fisher Scientific). We calculated LOH value as follows:

1-(smaller copy number from one allele / larger copy number from another allele) X100.

In our assay, LOH value never exceeds 10 at heterozygous SNP locus in normal tissue samples. We defined LOH-positive SNP when LOH values exceed 45. When a tumor exhibits LOH in at least one of three SNPs, we define the tumor as 9p24.2-LOH positive.

Statistics
All statistical analysis was completed using SPSS Software (Version 3, Chicago, IL). Logistic regression was used to investigate associations. Cox proportional hazard regression was used to determine risk factors for 5yr OS. In multivariate analysis, backward selection method (Backward LR: removes variables based on the probability of the likelihood-ratio) was used. Removal criteria were set to p=0.1. A p-value of less than 0.05 was deemed to be significant. Kaplan-Meier curve coupled with log-rank test was used for survival comparison. Chi-square test was used to determine relationships.

Data Availability
The data discussed in this study is available upon request from the corresponding author.

References

1. Satia JA, Keku T, Galanko JA, Martin C, Doctolero RT, Tajima A, Sandler RS, Carethers JM. Cancer Diet, lifestyle, and genomic instability in the North Carolina Colon Cancer Study. Epidemiol Biomarkers Prev. 2005;14(2):429-36.

2. Munakata K, Koi M, Kitajima T, et al. Inflammation-Associated Microsatellite Alterations Caused by MSH3 Dysfunction Are Prevalent in Ulcerative Colitis and Increase with Neoplastic Advancement. *Clin Transl Gastroenterol*. 2019;10(12):e00105. doi:10.14309/ctg.0000000000000105

3. Okita Y, Koi M, Takeda K, et al. Fusobacterium nucleatum infection correlates with two types of microsatellite alterations in colorectal cancer and triggers DNA damage. *Gut Pathog*. 2020;12(1):46. doi:10.1186/s13099-020-00384-3

4. Takeda K, Koi M, Okita Y, Sajibu S, Keku TO, Carethers JM. *Fusobacterium nucleatum* Load Correlates with *KRAS* Mutation and Sessile Serrated Pathogenesis in Colorectal Adenocarcinoma. *Cancer Research Communications*. 2023 Sep 26;3(9):1940-1951. doi: 10.1158/2767-9764.CRC-23-0179.

Supplemetary Table 1

Characterization of BA and WA Colon Cancer Cohort

Variables		No. of Cases (%) White	No. of Cases (%) Black	Univariate White vs Black O.R.	Univariate 95%CI	Univariate *p*	Multivariate White vs Black O.R.	Multivariate 95%CI	Multivariate *p*
Race		128	122	N.A.	N.A.	N.A.	N.A.	N.A.	N.A.
Age	≤65	55 (43)	70 (57.3)						
	>65	73 (57)	52 (42.7)	0.56	0.33-0.92	0.02	0.48	0.27-0.85	0.01
Sex	Female	50 (39)	66 (54.1)						
	Male	78 (61)	56 (45.9)	0.54	0.32-0.89	0.01	-	-	-
Tumor Locatior	Distal	48 (37.5)	48 (39.3)						
	Proximal	80 (62.5)	74 (60.7)	0.92	0.55-1.54	0.76	-	-	-
Tumor Stage	Local	43 (33.6)	42 (34.4)						
	Regional/Distant	85 (66.4)	80 (65.6)	0.96	0.57-1.62	0.89	-	-	-
MSI-H	No	108 (84.4)	117 (96)						
	Yes	20 (15.6)	5 (4)	0.23	0.08-0.63	0.005	0.25	0.08-0.75	0.01
EMAST /MSI-L	No	70 (54.7)	68 (55.7)						
	Yes	58 (45.3)	54 (44.3)	0.95	0.58-1.57	0.86	-	-	-
KRAS G12/G1:	No	84 (65.6)	67 (54.9)						
Mutations	Yes	44 (34.4)	55 (45.1)	1.56	0.91-2.61	0.08	-	-	-
BRAF V600E	No	115 (89.8)	113 (92.6)						
	Yes	13 (10.2)	9 (7.4)	0.7	0.29-1.71	0.44	-	-	-
9p24.2-LOH	No	90 (70.3)	105 (86.1)						
	Yes	38 (29.7)	17 (13.9)	0.38	0.2-0.72	0.003	0.39	0.19-0.78	0.008
Alcohol drink	No	75 (58.6)	98 (80.3)						
	Yes	53 (41.4)	24 (19.7)	0.34	0.19-0.61	<0.001	0.38	0.2-0.73	0.003
Smoking	Never	41 (32)	65 (53.2)						
	Current/Former	87 (68)	57 (46.8)	0.41	0.24-0.69	<0.001	0.53	0.3-0.94	0.03
Diabetes	No	103 (84.4)	92 (75.4)						
	Yes	25 (15.6)	30 (24.6)	1.34	0.73-2.45	0.33	-	-	-
High Blood Pre	No	75 (58.6)	50 (41)						
	Yes	53 (41.4)	72 (59)	2.03	1.23-3.37	0.006	2.46	1.35-4.49	0.003
Heart Problem	No	91 (71.1)	95 (77.9)						
	Yes	37 (28.9)	27 (22.1)	0.69	0.39-1.24	0.22	0.52	0.26-1.03	0.06

Association between race and other covariates was determined via univariate and multivariate logistic regression analysis.

In multivariate analysis, covariates were subjected to multiple backward selection untill all of the remaining covariates exhibit less than 0.1 p-value using SPSS software.

- : not included in final calculations

N.A.: not applicable
